# Controlled and uncontrolled asthma display distinct alveolar tissue matrix compositions

**DOI:** 10.1186/1465-9921-15-67

**Published:** 2014-06-20

**Authors:** Maria Weitoft, Cecilia Andersson, Annika Andersson-Sjöland, Ellen Tufvesson, Leif Bjermer, Jonas Erjefält, Gunilla Westergren-Thorsson

**Affiliations:** 1 Lung Biology Unit, Department of Experimental Medical Science, BMC, D12, Lund University, Lund, SE-221 84, Sweden; 2 Respiratory Medicine and Allergology, Department of Clinical Medical Sciences, Lund University, Lund, Sweden; 3 Unit of Airway Inflammation, Department of Experimental Medical Science, Lund University, Lund, Sweden

**Keywords:** Asthma, Controlled, Uncontrolled, ICS, Remodeling, Alveolar parenchyma, Extracellular matrix, Myofibroblasts

## Abstract

**Objective:**

Whether distal inflammation in asthmatics also leads to structural changes in the alveolar parenchyma remains poorly examined, especially in patients with uncontrolled asthma. We hypothesized that patients who do not respond to conventional inhaled corticosteroid therapy have a distinct tissue composition, not only in central, but also in distal lung.

**Methods:**

Bronchial and transbronchial biopsies from healthy controls, patients with controlled atopic and patients with uncontrolled atopic asthma were processed for immunohistochemical analysis of fibroblasts and extracellular matrix molecules: collagen, versican, biglycan, decorin, fibronectin, EDA-fibronectin, matrix metalloproteinase (MMP)-9 and tissue-inhibitor of matrix metalloproteinase (TIMP)-3.

**Results:**

In central airways we found increased percentage areas of versican and decorin in patients with uncontrolled asthma compared to both healthy controls and patients with controlled asthma. Percentage area of biglycan was significantly higher in both central airways and alveolar parenchyma of patients with uncontrolled compared to controlled asthma. Ratios of MMP-9/TIMP-3 were decreased in both uncontrolled and controlled asthma compared to healthy controls. In the alveolar parenchyma, patients with uncontrolled asthma had increased percentage areas of collagen, versican and decorin compared to patients with controlled asthma. Patients with uncontrolled asthma had significantly higher numbers of myofibroblasts in both central airways and alveolar parenchyma compared to patients with controlled asthma.

**Conclusions:**

Tissue composition differs, in both central and distal airways, between patients with uncontrolled and controlled asthma on equivalent doses of ICS. This altered structure and possible change in tissue elasticity may lead to abnormal mechanical properties, which could be a factor in the persistent symptoms for patients with uncontrolled asthma.

## Introduction

Asthma is a chronic inflammatory airway disease that is traditionally characterized by reversible central airway obstruction and airway hyperreactivity [1,2]. Although treatment with bronchodilators and inhaled glucocorticosteroids (ICS) normally provide good control of the disease, a significant proportion of the asthmatic patients have persistent symptoms despite conventional therapy [3]. This phenomenon, called uncontrolled asthma [4,5], represents a key challenge for increasing asthma control. Little is known about the inflammatory and remodeling processes causing persisting symptoms in this group of asthmatics. A likely cause of these symptoms is the presence of steroid-resistant components in both central and peripheral airways, as well as conventional inhalation therapy’s insufficient ability to reach the peripheral airways [6]. The peripheral airways have an important but poorly studied role in asthma pathophysiology [7,8]. The few previous studies that have studied transbronchial biopsies from patients with asthma provide clear indications that both small airways and alveolar tissues may be subjected to cellular inflammation [8-10].

In an inflammatory environment such as the asthmatic airway, fibroblasts are activated to myofibroblasts, which deposit molecules including collagens and proteoglycans into the surrounding extracellular matrix [11-13]. Myofibroblasts can be induced by a variety of factors e.g. transforming growth factor (TGF)-β_1_, the alternatively spliced domain A of fibronectin (EDA-fibronectin) as well as mechanical force [14]. Proteoglycans have several important functions in the tissue including storing cytokines such as TGF-β_1_, acting as structural components in tissue organization by forming large complexes with hyaluronan and collagens, as well as cell signaling affecting differentiation, migration and inflammation [15-17]. The matrix metalloproteinases (MMPs) are a family of proteases, involved in the breakdown of extracellular matrix [18]. A disruption in the balance between matrix production, MMPs and their regulators tissue inhibitors of metalloproteinases (TIMPs) can result in changes in extracellular matrix composition and fibrosis [19].

We have previously shown that increased collagen deposition occurs in alveolar parenchyma already in patients with mild untreated asthma [20]. However, how remodeling is linked to clinical control of asthma remains to be investigated. The aim of this study is to further advance this concept by a thorough investigation of extracellular matrix components and fibroblast densities in patients with controlled asthma and uncontrolled asthma on equivalent doses of ICS, compared to healthy controls. We hypothesized that patients with uncontrolled asthma have an altered tissue composition compared to both patients with controlled asthma and healthy controls, which could be one cause of the persistent symptoms in this group of patients.

## Methods

### Patient characteristics

The present study included 9 patients with controlled atopic asthma and 16 patients with uncontrolled atopic asthma according to GINA guidelines and asthma control test (ACT) [4,21]. Uncontrolled asthma was defined as still symptomatic despite treatment with ICS and beta-2 agonist (ACT score < 20). Eight healthy never-smoking non-atopic subjects (no respiratory symptoms, normal lung function, negative SPT and not hyper-responsive to methacoline) were used as controls. From each of the 33 subjects, central airway biopsies and transbronchial biopsies were collected during a study period from May 2007 to February 2012 at the Department of Respiratory Medicine, Lund University Hospital. All subjects gave their written informed consent to participating in the study, which was approved by the ethics committee in Lund (LU213-05). For details on the skin prick test, exhaled NO measurements, sputum induction and processing, spirometry and methacoline inhalation challenge test, see Additional file 1.

### Bronchoscopy with collection of bronchial and transbronchial biopsies and bronchoalveolar lavage

From each patient, central airway biopsy specimens (n = 5) were taken from the segmental or subsegmental carina in the right lower and upper lobes, followed by sampling of transbronchial biopsy specimens (n = 5) in the right lower lobe. In total, 330 biopsies were collected. Bronchoscopy was performed as previously described [8]. Bronchoalveolar lavage (BAL) was performed in the right middle lobe. For details see Additional file 1.

### Processing of tissue

For processing of tissue, see Additional file 1. Sections were pre-treated and deparaffinized in an automated PT link (Dako, Glostrup, Denmark) according to Additional file 1: Table S2.

### Immunohistochemistry

All antibodies used have been extensively validated for staining of human tissue in research and routine clinical diagnosis (Additional file 1: Table S2). Staining was absent in sections using isotype-matched control antibodies (Dako, Glostrup, Denmark). For trichrome staining, see Additional file 1.

#### Immunohistochemical staining of versican, decorin, biglycan, fibronectin, EDA-fibronectin, MMP-9 and TIMP-3

A double staining protocol (EnVision™ G|2 Doublestain System, K5361, Dako) was used for simultaneous visualization of versican and decorin. A single staining protocol (EnVision™ Detection system, K5007, Dako) was used for visualization of biglycan, fibronectin, EDA-fibronectin, MMP-9 and TIMP-3. The immunohistochemistry protocols were performed using an automated immunohistochemistry robot (Autostainer, Dako). Sections were stained with Mayer’s haematoxylin for visualisation of background tissue and mounted. For details see Additional file 1.

### Tissue analysis

#### Quantification of versican, decorin, biglycan, fibronectin, EDA-fibronectin, MMP-9 and TIMP-3

Stained slides were digitally scanned using ScanScope (Aperio, Vista, CA). All markers were quantified in blinded sections in central airways (bronchial biopsies) and alveolar parenchyma (transbronchial biopsies). The immunoreactivity (% positively stained area) of versican, decorin, biglycan, fibronectin, EDA-fibronectin, MMP-9 and TIMP-3 as well as the tissue area in the walls of bronchi and in the alveolar septa was calculated using Visiomorph™ (Visiopharm, Hoersholm, Denmark). The percentage stained area was calculated dividing the stained area by the total selected area. The image analysis program calculated the tissue area of the whole biopsy, excluding air spaces so that only tissue (i.e. airway epithelium, lamina propria and smooth muscle layer, or the alveolar septa) was measured. Glands were excluded from the analysis by manual detection.

#### Quantification of myofibroblasts

The density of fibroblasts and myofibroblasts was counted manually and calculated either as double positive for prolyl 4-hydroxylase (P4OH) and vimentin (fibroblasts) or triple positive for αSMA, P4OH and vimentin (myofibroblasts). The number of cells was related to tissue area (cells/mm^2^) using Visiomorph™ (Visiopharm). For details see Additional file 1.

### Immunoassay for MMP-9 in BAL and sputum

Concentration of MMP-9, in bronchoalveolar lavage (BAL) fluid and sputum samples was measured by enzyme-linked immunosorbent assay (ELISA) (MMP-9 ELISA DuoSet, R&D Systems, MN, USA).

### Statistical analysis

Data are presented as median values together with interquartile range unless otherwise stated. Non-parametric Mann–Whitney was used for comparison between two groups with Bonferroni post hoc test for correction of multiple comparisons. Correlation analysis was performed using Spearman rank correlation test. Critical p values (alpha) were adjusted according to Bonferroni. Subsequently, alpha values of <0.017 (*), <0.0033 (**) and <0.00033 (***) denote significant levels of difference. All analysis was performed using GraphPad Prism version 6.0a software (GraphPad Software, San Diego USA).

## Results

### Demographic and clinical characteristic

An overview of patient characteristics is presented in Table 1.

**Table 1:** Subject characteristics

	**Healthy controls**	**Controlled asthma**	**Uncontrolled asthma**
Subjects, n	8	9	16
Gender, men/women	3/5	6/3	10/6
Age, years	23 [22–28]	29 [25–35]	44 [28–52]
FEV_1_, (L)	3.7 [3.4–5.1]	4.0 [3.3–4.8]	3.0 [2.4–4.0]
FEV_1_, % of predicted	98 [91–113]	91 [86–103]	81 [72–88]
PD_20_, (μg)	> 2000	296 [74–1143]	266 [96–531]
FeNO_50_, (ppb)	15 [9–17]	24 [17–36]	28 [20–48]
Bronchial NO, (ppb)	0.75 [0.45–0.98]	1.2 [0.9–1.6]	1.4 [0.85–2.7]
Alveolar NO, (ppb)	2.1 [2–2.5]	2.7 [1.7–3.4]	2.7 [1.8–3.5]
Atopy, yes/no	0/8	9/0	16/ 0
Rhinitis, yes/no	0/8	9/0	15/ 1
ICS/day, (μg)	-	800 [400–800]	800 [400–800]
Smoking, yes/no	0/8	0/9	0/16
ACT score	-	23 [22–24]	17 [13–19]

*FEV*_*1*_ = forced expiratory volume in 1 second, *PD*_*20*_ = provocative dose (methacholine) producing a fall in FEV_1_ of 20 %, *FeNO*_*50*_ = exhaled nitric oxide, measured at a flow rate of 50 ml ⁄ s, *NO* = nitric oxide, *ppb* = parts per billion, *ICS* = inhaled glucocorticosteroids, *ACT* = asthma control test.

#### Uncontrolled asthma

Two patients were treated with leukotriene-receptor antagonist; three patients were treated with antihistamines and two with nasal steroids. FEV_1_ % predicted was lower in patients with uncontrolled asthma compared to healthy controls (p = 0.018). FeNO was higher in patients with uncontrolled asthma compared to controls (p = 0.001). There was no significant difference in alveolar NO between patients with uncontrolled asthma and controls.

#### Controlled asthma

There was no difference in FEV_1_ % predicted or exhaled NO between healthy controls and patients with controlled asthma. All asthmatics were treated with inhaled glucocorticosteroids and inhaled bronchodilators. All asthmatics were non-smokers although three were ex-smokers.

### Characterization of connective tissue alterations in patients with controlled or uncontrolled asthma and healthy controls

#### Increased percentage area of collagen in alveolar parenchyma of patients with uncontrolled, but not controlled asthma

**Figure 1:**
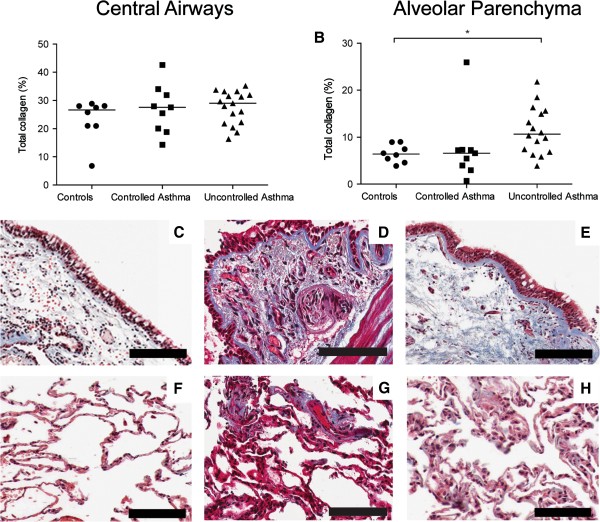
**Percentage area of collagen (trichrome staining, % positively stained area) in bronchial and transbronchial biopsies from controls and patients with controlled and uncontrolled asthma (A-B).** Data are presented as scatter dot plots where horizontal line denotes median value. Representative micrographs of tissue stained with trichrome staining (collagen: blue) from controls **(C, F)**, controlled asthma **(D, G)** and uncontrolled asthma **(E, H)**. Scale bars: **C-E, G** and **H** = 100 μm and **F** = 200 μm.

Masson’s trichrome staining was used to examine total collagen content (Figure 1). In the central airways, collagen was seen in the subepithelial region (i.e. the lamina propria) and reticular basement membrane (Figure 1C-E). In the transbronchial biopsies, collagen was found in the septa of the alveolar parenchyma (Figure 1F-H). The alveolar parenchyma displayed an increased percentage area of collagen in patients with uncontrolled asthma compared to healthy controls (p = 0.011), Figure 1B.

#### Increased percentage area of versican and decorin but not biglycan in patients with uncontrolled asthma

**Figure 2:**
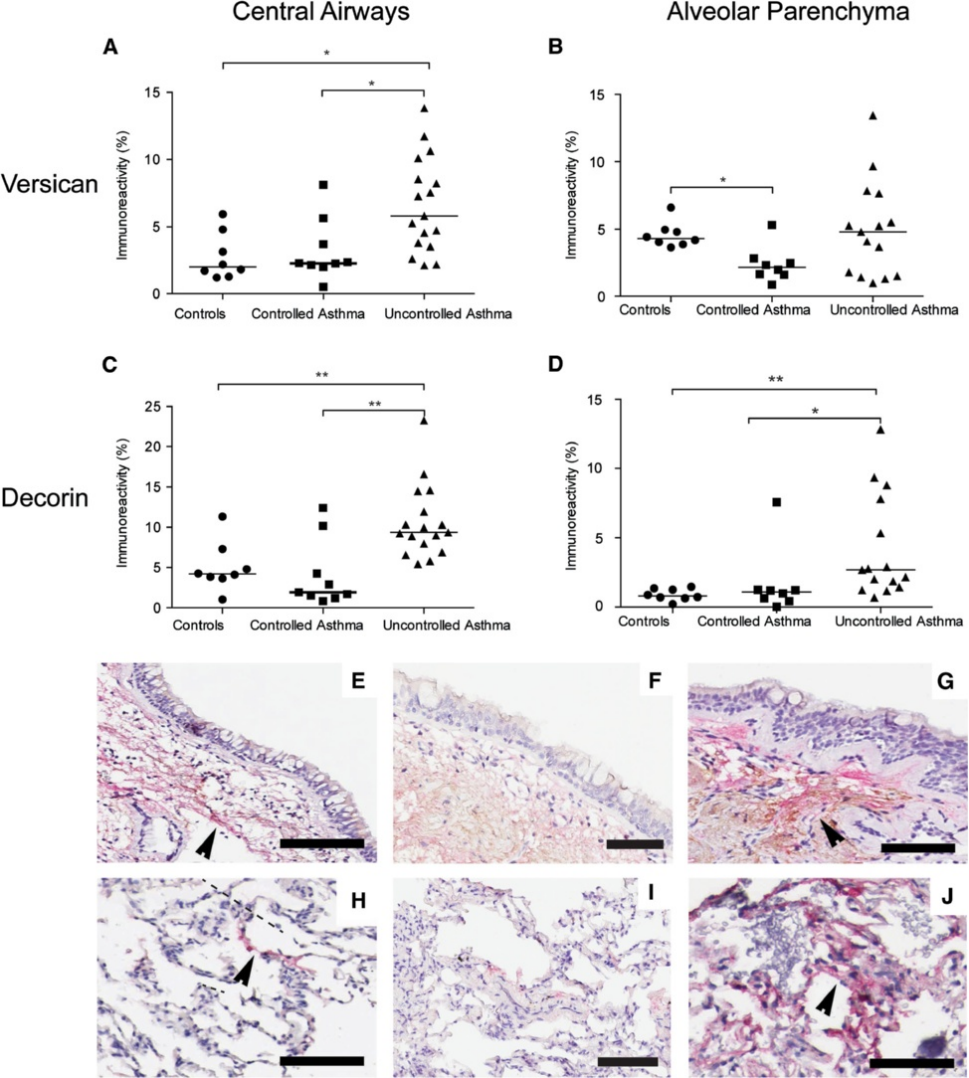
**Percentage area of versican (A, B) and decorin (C, D) (% positively stained area) in bronchial and transbronchial biopsies from controls and patients with controlled and uncontrolled asthma.** Data are presented as scatter dot plots where horizontal line denotes median value. Representative micrographs of double staining of versican (brown) and decorin (red) from controls **(E, H)** and patients with controlled asthma **(F, I)** and uncontrolled asthma **(G, J)** in bronchial **(E-G)** and transbronchial **(H-J)** biopsies. Scale bars: **F, G** = 50 μm, **E, I** and **J** = 100 μm, **H** = 200 μm.

**Figure 3:**
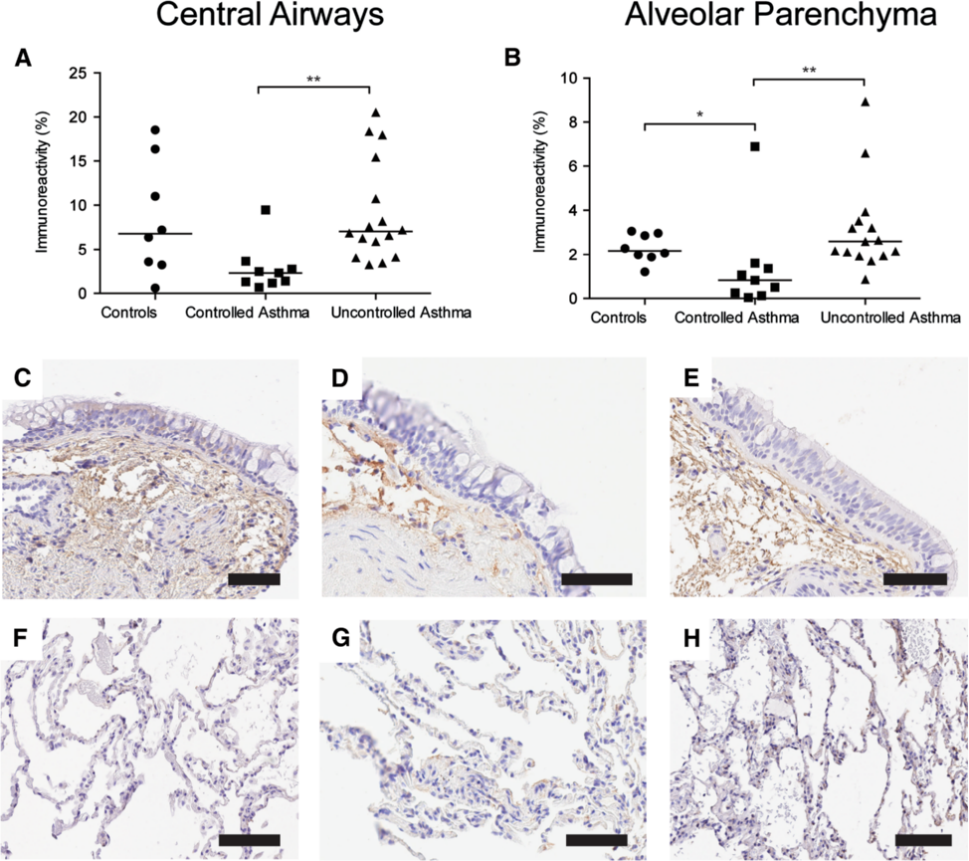
**Percentage area of biglycan (% positively stained area) in bronchial (A) and transbronchial biopsies (B) from controls and patients with controlled and uncontrolled asthma.** Representative micrographs of staining of biglycan (brown) from controls **(C, F)** and patients with controlled asthma **(D, G)** and uncontrolled asthma **(E, H)** in bronchial **(C-E)** and transbronchial **(F-H)** biopsies. Scale bars: **C-H** = 50 μm.

In central airways, versican and decorin expression was seen in elastic fibers in the lamina propria (Figure 2E-G). Decorin expression was located adjacent to the reticular basement membrane while versican predominantly was found deeper in the airway wall closer to the smooth muscle bundles. In the alveolar parenchyma, versican and decorin expression was found in irregular and patchy areas in the alveolar septa (Figure 2H-J). Percentage area of versican was increased in central airways in patients with uncontrolled asthma compared to both patients with controlled asthma (p = 0.011) and healthy controls (p = 0.0038), Figure 2A. However, in the alveolar parenchyma there was a decreased percentage area of versican in patients with controlled asthma compared to healthy controls (p = 0.007), Figure 2B.In central airways, decorin was increased in patients with uncontrolled asthma compared to both patients with controlled asthma (p = 0.0028) and healthy controls (p = 0.0015), Figure 2C. In the alveolar parenchyma, percentage area of decorin was significantly higher in patients with uncontrolled asthma compared to healthy controls and patients with controlled asthma (p = 0.0014 and p = 0.0067 respectively), Figure 2D.Biglycan expression was seen in elastic fibers in the lamina propria of the central airway wall and in irregular and patchy areas in the alveolar septa. In central airways, percentage area of biglycan was decreased in patients with controlled asthma compared to patients with uncontrolled asthma (p = 0.0004), Figure 3A and C-E. The same pattern was seen in the alveolar parenchyma, where the percentage area of biglycan was decreased in patients with controlled asthma compared to healthy controls and patients with uncontrolled asthma (p = 0.011 and p = 0.0017 respectively), Figure 3B and F-H.

#### Decreased MMP-9 and increased TIMP-3 in central airways and alveolar parenchyma of patients with controlled and uncontrolled asthma

**Figure 4:**
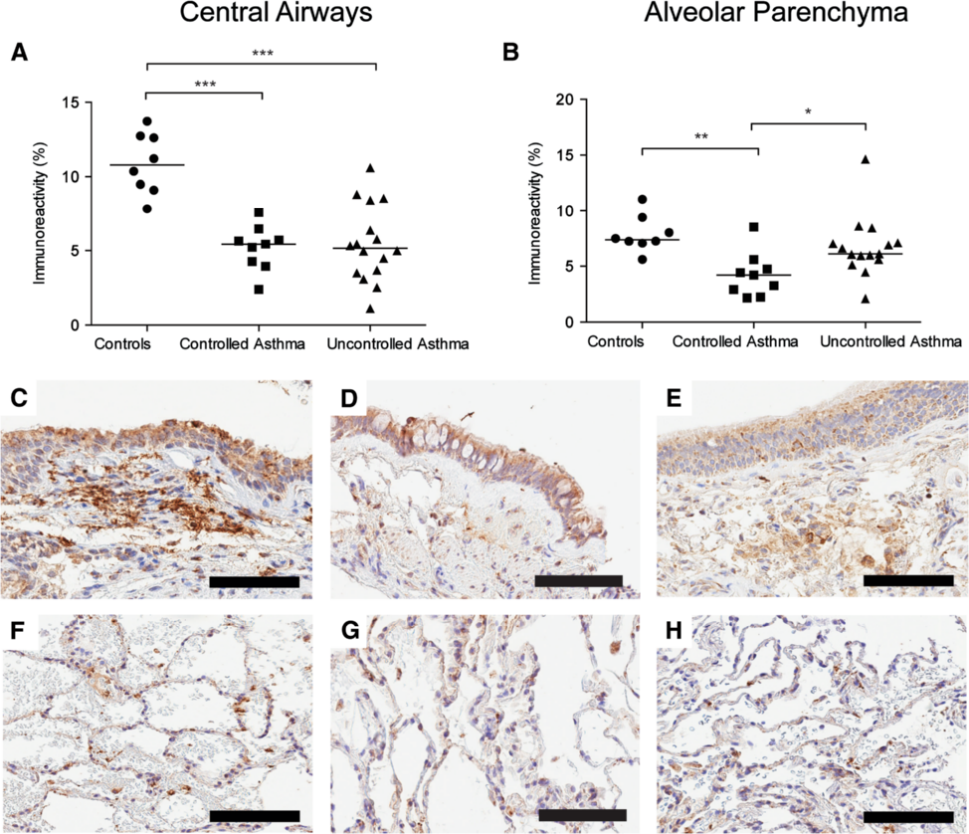
**Percentage area of MMP-9 (% positively stained area) in bronchial (A) and transbronchial biopsies (B) in controls and patients with controlled and uncontrolled asthma.** Representative micrographs of staining of MMP-9 (brown) from controls **(C, F)** and patients with controlled asthma **(D, G)** and uncontrolled asthma **(E, H)** in bronchial **(C-E)** and transbronchial **(F-H)** biopsies. Scale bars: **C-E** and **G** = 100 μm, **F** and **H** = 200 μm.

**Figure 5:**
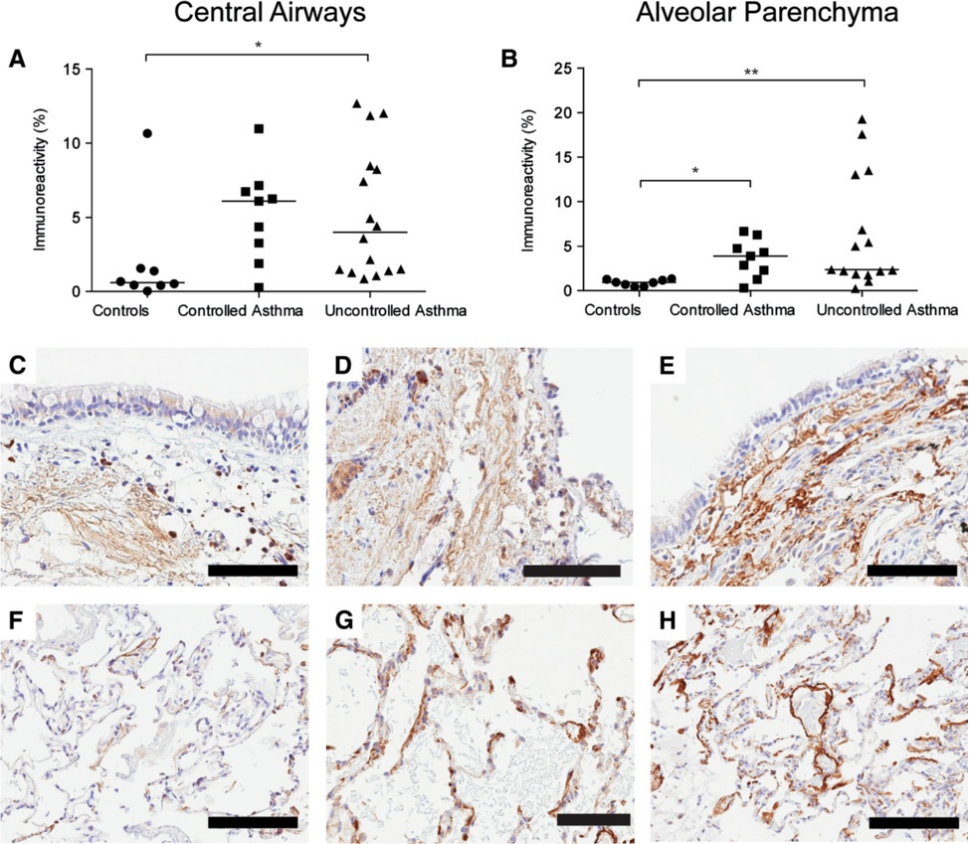
**Percentage area of TIMP-3 (% positively stained area) in bronchial (A) and transbronchial biopsies (B) in controls and patients with controlled and uncontrolled asthma.** Representative micrographs of staining of TIMP-3 (brown) from controls **(C, F)** and patients with controlled asthma **(D, G)** and uncontrolled asthma **(E, H)** in bronchial **(C-E)** and transbronchial **(F-H)** biopsies. Scale bars: **C-E** and **G** = 100 μm, **F** and **H** = 200 μm.

Expression of MMP-9 was primarily seen in columnar cells in the airway epithelium and in inflammatory cells in the lamina propria and adventitia of the central airway wall and in cells located in the alveolar septa, Figure 4. Double immunohistochemical staining showed that MMP-9 positive cells were mainly neutrophils, macrophages and scattered mast cells, Additional file 1: Figure S3. A constitutive low intensity expression of MMP-9 was found in both healthy controls and patients with asthma. However, scattered strong positive epithelial cells and inflammatory cells were predominantly found in controls. Expression of TIMP-3 was found in the elastic fibers of the lamina propria of the central airway wall as well as in the matrix surrounding the smooth muscle bundles, Figure 5A and C-E. In the alveolar parenchyma TIMP-3 expression was found in irregular and patchy areas in the alveolar septa, Figure 5B and F-H. The percentage area of MMP-9 was decreased in central airways of patients with controlled and uncontrolled asthma compared to healthy controls (p < 0.0001 and p = 0.0001 respectively), Figure 4A and E-C. There was also a decreased percentage area of MMP-9 in the alveolar parenchyma of patients with controlled asthma compared to healthy controls and patients with uncontrolled asthma (p = 0.0025 and p = 0082 respectively), Figure 4B and F-H.

In central airways, there was an increased percentage area of TIMP-3 in patients with uncontrolled asthma compared to healthy controls (p = 0.087), Figure 5A and C-E. There was also an increased percentage area of TIMP-3 in the alveolar parenchyma, in both patients with controlled and uncontrolled asthma compared to healthy controls (p = 0.011 and p = 0.0008 respectively), Figure 5 B and F-H. For MMP-9/TIMP-3 ratios, see Additional file 1: Figure S3. There was no significant difference in MMP-9 concentration in BAL or sputum between healthy controls and patients with asthma, Additional file 1: Figure S2. For cellular profile in BAL, see Additional file 1: Table S1.

#### Increased numbers of myofibroblasts in alveolar parenchyma of patients with uncontrolled asthma

**Figure 6:**
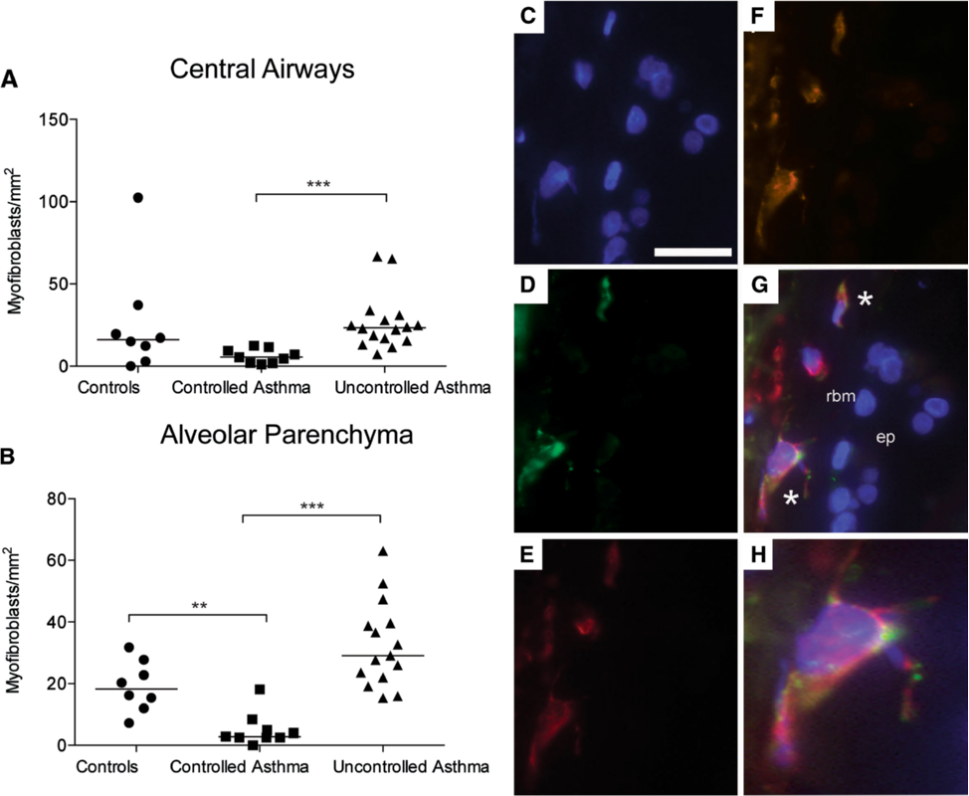
**Density of myofibroblasts per tissue area (triple positive cells/mm**^**2**^**) in central airways (A) and alveolar parenchyma (B) from controls and patients with controlled and uncontrolled asthma.** Representative micrographs of triple staining of myofibroblasts: **(C)** nuclei: Hoechts 33342, blue, **(D)** prolyl-4OH: Alexa F488, green, **(E)** vimentin: AlexaF 647, deep red, **(F)** α-SMA: AlexaF 555, red. Merged image are shown in **(G)** where * denotes triple positive cells. High magnification image of fibroblast with protrusions are shown in **(H)**. rbm: reticular basement membrane and ep: airway epithelium

Triple positive myofibroblasts (αSMA, P4OH and vimentin) were found in central airway wall, predominantly situated just below the reticular basement membrane. In central airways, patients with controlled asthma had significantly fewer myofibroblasts than patients with uncontrolled asthma (p < 0.0001), Figure 6A. No difference in myofibroblast numbers was found between patients with uncontrolled asthma and healthy controls in the central airways. Scattered myofibroblasts were found in the septa of the alveolar parenchyma. In the alveolar parenchyma, patients with controlled asthma had significantly fewer myofibroblasts than healthy controls and patients with uncontrolled asthma (p = 0.0016 and p < 0.0001 respectively), Figure 6B. In patients with controlled asthma the number of myofibroblasts correlated negatively with ICS-dose per day (r_s_ = −0.75, p = 0.008).

#### Tendency towards increased percentage area of EDA-fibronectin in alveolar parenchyma of patients with uncontrolled asthma but not in patients with controlled asthma

**Figure 7:**
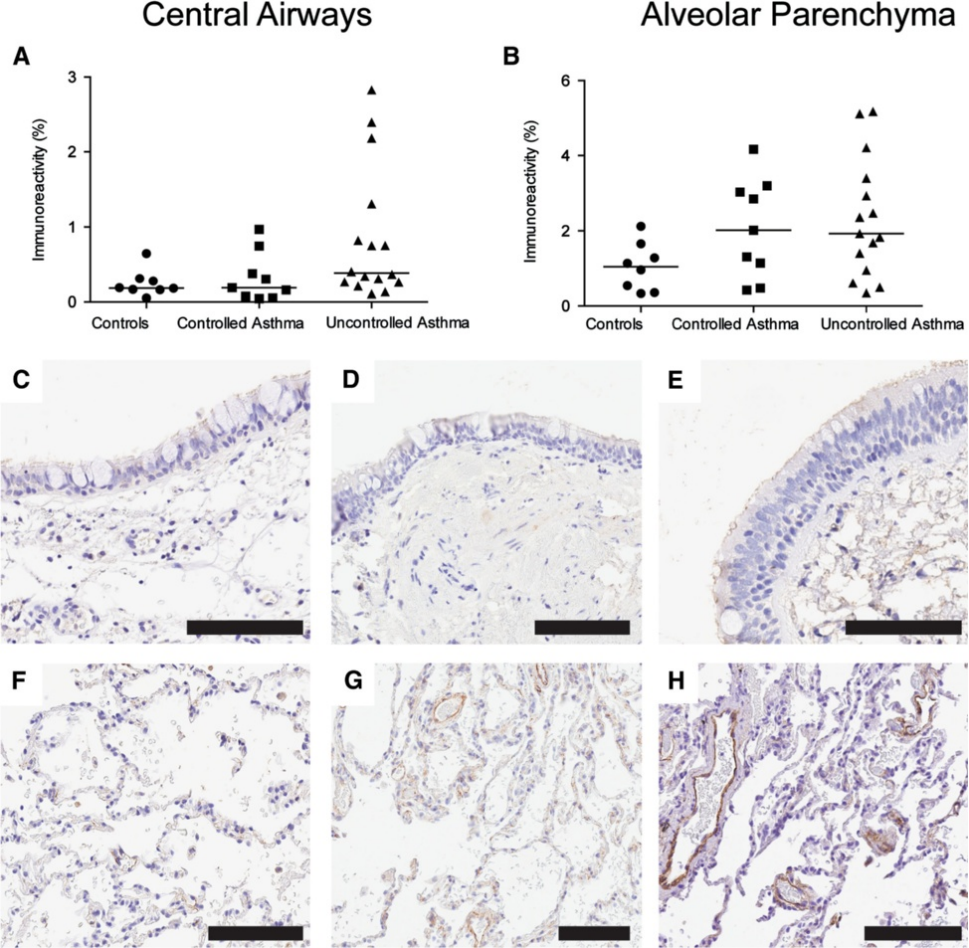
**Percentage area of EDA-fibronectin (% positively stained area) in controls and patients with uncontrolled asthma (F).** Representative micrographs of staining of EDA-fibronectin (brown) from controls **(C, F)** and patients with controlled asthma **(D, G)** and uncontrolled asthma **(E, H)** in bronchial **(C-E)** and transbronchial **(F-H)** biopsies. Scale bars: **C-H** = 100 μm.

Expression of fibronectin and EDA-fibronectin was measured in central airways and alveolar parenchyma in patients with controlled and uncontrolled asthma and healthy controls. No difference in total fibronectin percentage area was found between healthy controls and asthmatics in either central airways or alveolar parenchyma, Additional file 1: Figure S4. There was a tendency towards increased percentage area of alternatively spliced domain A of fibronectin (EDA-fibronectin) in central airways and alveolar parenchyma, of patients with uncontrolled asthma compared to healthy controls, Figure 7A-H.

## Discussion

This study shows that the bronchial wall and alveolar parenchyma have an altered composition of matrix that differs between patients with controlled and uncontrolled asthma on equivalent doses of ICS, which could contribute to the symptoms in patients with uncontrolled asthma.

Due to the inaccessibility of tissue from alveolar parenchyma of asthmatic patients little is known regarding the remodeling processes in the peripheral lung and how these changes contribute to asthma pathology [20]. The present study applied a unique study design of obtaining bronchial as well as transbronchial biopsies from patients with uncontrolled and controlled asthma on equivalent doses of ICS, as well as from healthy control subjects. Emerging new evidence show that distal airways are subjected to an inflammatory process in asthmatic patients [8,10]. TGF-β_1_ plays a central role in airway remodeling and has been found to induce production of several types of collagens from myofibroblasts [22,23]. Although the patients with uncontrolled asthma had significantly thicker reticular basement membrane [24] there was no difference in total collagen content in central airways when measuring the whole biopsy. However, expression of collagen in distal airways was increased in uncontrolled asthmatics but not in patients with controlled asthma, compared to controls. Thickening of alveolar walls due to increased amounts of collagen may lead to impaired gas exchange in the alveoli. We have previously reported on increased collagen in distal airways of patients with mild untreated asthma [20], which raises the question if this accumulation of collagen could be decreased with ICS treatment and also why the patients with uncontrolled asthma have increased areas of collagen despite treatment. The patients were determined to be uncontrolled at the time when the biopsies were taken - could an increased dose of ICS reverse this accumulation of collagen?

Biglycan and decorin are two small leucine-rich proteoglycans involved in collagen fibrillogenesis and fibril stabilization in the tissue [25]. Both decorin and biglycan have been found to bind TGF-β_1_*in vitro*[26]. However, a study by Kolb *et al.* showed that, *in vivo*, only decorin and not biglycan was found to interfere with TGF-β_1_ activity [27]. In addition, decorin has been found to act protective against liver fibrosis by attenuating TGF-β_1_ signaling [28]. In this aspect, one can speculate that the increase in decorin expression in patients with uncontrolled asthma could be a protective mechanism to mitigate remodeling. On the other hand, increased amounts of decorin could by regulating cross-linking and interfibrillar spacing create a stiffer collagen matrix, which could affect the overall elasticity of the lung tissue. Furthermore, decorin could also have a role in protecting collagen fibrils from cleavage by collagenases, as *in vitro* studies of collagenases has shown [29], which could contribute to the accumulation of collagen in the tissue. While the quantity of proteoglycan present is partly responsible for collagen fibril morphology, variations in the glycosaminoglycan chains have also been shown to play important roles in e.g. collagen fibril size control and interfibrillar spacing [30-32]. Characterization of glycosaminoglycan chains from isolated biglycan and decorin from asthmatics and healthy individuals could provide valuable information on the subject.

Versican is a large proteoglycan that is highly interactive with several matrix components including hyaluronan and fibrillin [33,34]. Increased levels of versican have been reported in diseases such as atherosclerosis and cancers [35,36]. Accumulation of versican, as seen in the central airways of patients with uncontrolled asthma, could also lead to increased stiffness around cells, which in turn can influence their ability to migrate, proliferate, adhere and remodel the matrix [37]. Versican could also increase stiffness in the lung by inhibiting elastin-binding proteins and interfering with the assembly of elastic fibers, thereby affecting lung function [38]. We can only speculate regarding the decreased percentage areas of biglycan and versican in controlled asthmatics. In cultured fibroblasts serum induced production of proteoglycans is reduced by addition of corticosteroids [39], since the fibroblasts used in this study are “healthy”, this could be in accordance with how fibroblasts in patients with controlled asthma respond to corticosteroids. It is possible that there is a difference in fibroblast response between the controlled and uncontrolled asthmatics, or that the inhaled corticosteroids do not reach the peripheral airways.

There was a decreased ratio between MMP-9 and TIMP-3, in central and distal airways of both patient groups, which indicates a proteolytic-antiproteolytic imbalance. Although, since the ratio is decreased in both patient groups it does not explain the differences in percentage areas of matrix molecules between patients with controlled and uncontrolled asthma. Additional analyses of other MMPs, TIMPs and their relationship with structural alterations in the lung tissue need to be further examined. Taken together, these structural alterations affect phenotypes of cells and the biomechanical properties of the lung.

The fibroblasts/myofibroblasts play a pivotal role in remodeling processes as the main extracellular matrix producing cells. Corticosteroids have been found to prevent myofibroblast accumulation and airway remodeling in mice [40]. Interestingly, we found increased numbers of myofibroblasts in alveolar parenchyma in patients with uncontrolled asthma compared to healthy controls while the number of myofibroblasts was decreased in patients with controlled asthma compared to both controls and patients with uncontrolled asthma. Levels of TGF-β_1_ mRNA as well as immunoreactivity have been found to be increased in the airways submucosa of asthmatic patients, with a direct correlation to the severity of the disorder [41]. Interleukin-13 (IL-13) is another important mediator in asthma pathogenesis, which has been associated with tissue fibrosis, both by inducing proliferation of fibroblasts and collagen production but also by activation of TGF-β_1_[42-44].

EDA-fibronectin cooperates with TGF-β_1_ in modulating fibroblasts into the activated myofibroblast phenotype [14]. The increased amount of myofibroblasts in the alveolar parenchyma of patients with uncontrolled asthma could be linked to the increased expression of EDA-fibronectin, and is in accordance with our finding of an increased percentage area of several matrix components such as collagen and decorin.

The differences in myofibroblast numbers, in patients with controlled asthma compared to uncontrolled asthma, raises the question how the ICS treatment affects the tissue and why it varies between patient groups. Could it be due to differences in steroid-sensitivity, or does the inhalation therapy not reach the peripheral airways? Indeed, in patients with controlled asthma there was a negative correlation between numbers of myofibroblasts and daily dose of ICS, which was not found in the group of uncontrolled asthmatics. Although ICS has a well-documented effect on airway inflammation, little is known regarding long-term effects on remodeling and mesenchymal cell growth. Effects of steroids on extracellular matrix seem to depend on the nature of the study since *in vitro* and *in vivo* animal and human studies often show conflicting results. This matter is further complicated by the possibility that myofibroblasts respond differently to steroids in some patient-groups which is concordant with the heterogeneity of asthma.

### Summary

Our study shows that tissue composition differs between patients with controlled and uncontrolled asthma on equivalent doses of ICS. Our data support the notion that patients who have remaining symptoms despite conventional ICS therapy have a pronounced matrix remodeling in both central and distal airways and should thus benefit from treatment strategies that target specific inflammatory and remodeling responses in the distal lung. The increased percentage areas of several matrix components and myofibroblasts could be a contributing factor to the persistent symptoms in uncontrolled asthma. Due to the heterogeneity of the disease, asthma medicine needs to be individualized, and new treatments for prevention of remodeling needs to be developed. Currently, several therapies directed at IL-13 are in development [45]. How those therapies and other systemic treatments like oral corticosteroids, Omalizumab (Anti-IgE) and Mepolizumab (Anti-IL-5) etc. affect the inflammatory response, and structural changes in the peripheral lung, is an important future research area.

## Abbreviations

ACT: Asthma control test; BAL: Bronchoalveolar lavage; ICS: Inhaled glucocorticosteroids; TGF-β: Transforming growth factor-β; SPT: Skin-prick test; GAG: Glycosaminoglycan; MMP: Matrix metalloproteinase; TIMP: Tissue-inhibitor of matrix metalloproteinase.

## Competing interest

None of the authors has a financial relationship with a commercial entity that has an interest in the subject of the presented manuscript or other competing interests to disclose.

## Authors’ contributions

MW: Contributed to the study design, performed laboratory work, quantified immunostainings, performed statistical analysis, interpreted the data and wrote the manuscript. CA: Contributed to the study design, performed laboratory work, interpreted the data and critically revised the manuscript. AAS: Interpretation of data and revision of the manuscript. ET: Contributed with clinical characterization of the patients, performed MMP-9 ELISA on sputum and revised the manuscript. LB: Recruited patients, collected materials, supervised the study and revised the manuscript. JE and GWT: Contributed to study design, supervised the study, interpreted data and critically revised the manuscript. All authors approved the final version of the manuscript.

## Supplementary Material

Additional file 1 Online supplement Click here for file
