# Supplementary material for: Controlled and uncontrolled asthma display distinct alveolar tissue matrix compositions
Source: Respir Res. 2014 Jun 20;15(1):67. doi: 10.1186/1465-9921-15-67 (PMC4089934; doi:10.1186/1465-9921-15-67)
Supplement: Additional file 1 — Online supplement [file 1465-9921-15-67-S1.docx]

**Additional file 1**

**Controlled and Uncontrolled Asthma Display Distinct Alveolar Tissue Matrix Compositions**

Maria Weitoft^a^, MSc, Cecilia Andersson^b^, PhD, Annika Andersson-Sjöland^a^, PhD, Ellen Tufvesson^b^, PhD, Leif Bjermer^b^, MD, PhD, Jonas Erjefält^a^, PhD and Gunilla Westergren Thorsson^a^, PhD.

*From the ^a^Lung Biology Unit, Department of Experimental Medical Science and the ^b^ Respiratory Medicine and Allergology, Department of Clinical Medical Sciences, Lund University, Lund Sweden*

Corresponding author: Maria Weitoft, MSc, Experimental Medical Science, BMC, D12, Lund University, SE-221 84 Lund, Sweden. Telephone: +46 -46-222-94-41. Fax: +46-46-222 45-46. E-mail address: maria.weitoft@med.lu.se

Funding: This study was supported by the Swedish Medical Research Council (11550), Stockholm Sweden, the Evy and Gunnar Sandberg foundation, Lund, Sweden, the Heart-Lung Foundation, Stockholm, Sweden, Greta and John Kock, Trelleborg, Sweden, the Alfred Österlund Foundation, Malmö, Sweden, the Anna-Greta Crafoord Foundation, Stockholm, Sweden, the Konsul Bergh Foundation, Stockholm, Sweden, the Royal Physiographical Society in Lund, Sweden and the Medical Faculty of Lund University, Sweden.

None of the authors has a financial relationship with a commercial entity that has an interest in the subject of the presented manuscript or other conflicts of interests to disclose.

**METHODS**

**Allergy Screening**

Standardized skin prick test (SPT) (Alk Abello, Copenhagen, Denmark) was performed on all subjects. Atopy was defined as a positive SPT (weal bigger than or equal to histamine positive control) to one or more airborne allergens included in the test panel (birch, timothy, mugwort, cat, dog, horse, *D. pteronyssinus, D. farinae, Aspergillus fumigatus* and *Cladosporium herbarum*) (ALK Abello, Copenhagen, Denmark). For subjects with positive SPT to pollen, bronchoscopy procedure was performed outside pollen season.

**Sputum Induction and Processing**

Briefly, sputum was induced by inhalation (0.5, 1, 2 and 4 min) of nebulized isotonic saline solution (0.9% NaCl) followed by a hypertonic solution (4.5% NaCl). Lung function was measured 1 min after each induction time-point. Subjects were asked to rinse their mouth and blow their nose, and try to cough between each dose of nebulized saline [[1](#_ENREF_1)]. Sputum plugs were sorted out, and treated with four volumes of 0.65 mM dithiothreitol (DTT) in phosphate-buffered saline (PBS) for 1h in +4**°**C. Additional 4 volumes of PBS was added, followed by filtration through a 60 μm filter and a final centrifugation (1000 g for 5 min), which separated the supernatant from the cells.

**Bronchoscopy with Collection of Bronchial and Transbronchial Biopsies and Bronchoalveolar Lavage**

Bronchoscopy was performed as previously described [[2](#_ENREF_2)]. From each patient, central airway biopsy specimens (n=5 per patient) were taken from the segmental or subsegmental carina in the right lower and upper lobes, followed by sampling of transbronchial biopsy specimens (n=5 per patient) in the right lower lobe. In total, 330 biopsies were collected. After local anaesthesia with a flexible bronchoscope (Olympus IT160, Tokyo, Japan) and transbronchial biopsy specimens were taken with biopsy forceps (Olympus FB211D) under fluoroscopic guidance in the peripheral right lower lobe at a distance of >2 cm from the chest wall. Fluoroscopy was performed immediately and 2 h after the procedure to rule out significant bleeding or pneumothorax. Bronchoalveolar lavage (BAL) was performed by flushing the airways with up to 3x50 ml of 0.9 M NaCl, and the resulting fluid was used for analysis. Cells in BAL were collected using a cytocentrifuge (Shandon Southern Products Ltd, Runcorn, Cheshire, UK) at 1500 rpm for 5 minutes. Differential cell counts of BAL fluid were performed on cytospin preparations stained by May-Grünewald-Giemsa method. In total, 600 cells/patient were counted, see results in Table SI.

**Exhaled NO measurements**

Measurements were performed as previously described [[3](#_ENREF_3)]. Briefly, FeNO measurements were done prior to bronchial challenge testing at a flow rate of 50, 100, 200, 300 and 400 ml/s using a NIOX NO analyser (Aerocrine AB, Stockholm, Sweden), and the results were expressed as parts per billion (ppb). Alveolar NO concentration and bronchial flux of NO were calculated with a two-compartment linear model using a flow rate of 100-400 ml/s [[4](#_ENREF_4)].

**Spirometry and Methacoline Inhalation Challenge Test**

For measurements of lung function and a MasterScope spirometer (v. 4.5, Erich Jaeger GmbH, Wurzburg, Germany) was used with reference values from Crapo [[5](#_ENREF_5)]. Only patients with a FEV_1_ baseline value of ≥70 % of predicted were included in the study. Presence of bronchial hyper-responsiveness was measured with a methacoline challenge test, (Aerosol Provocation System, APS; Erich Jaeger GmbH) as described previously [[6](#_ENREF_6)]. A positive test was defined as the cumulative dose that caused a decline in FEV1 by 20% or more from baseline.

**Processing of Tissue**

Bronchial and transbronchial biopsies were immediately placed in 4% buffered formaldehyde (fixation time: 1.5-2 hours at room temperature and 15-19 hours at 4°C), dehydrated and embedded in paraffin and sequential 3 µm sections were generated. Serial sections from all paraffin blocks were stained with Mayer’s haematoxylin and these were used to select 2 bronchial and 2 transbronchial biopsies from each patient that had a well-preserved morphology and were without any crush, or mechanically-induced stretch artefacts. Before immunohistochemical staining, sections were pre-treated and deparaffinised in an automated PT link (Dako, Glostrup, Denmark) according to Table SII. For staining without pre-treatment, sections were deparaffinised in xylene and rehydrated in decreasing concentrations of alcohol.

**Immunohistochemistry**

All antibodies used have been extensively validated for staining of human tissue in research and routine clinical diagnosis (Table SII). Staining was absent in sections using isotype-matched control antibodies (Dako, Glostrup, Denmark).

*Trichrome Staining*

Paraffin sections were stained with Masson’s trichrome for measurement of collagen expression. Masson’s trichrome staining is a three-colour staining protocol were collagen fibers will be stained blue, nuclei will be stained black and the background is stained red.

*Immunohistochemical Staining of Versican, Decorin, Biglycan, MMP-9, TIMP-1 and -3*

A double staining protocol (EnVision™ G|2 Doublestain System, K5361, Dako) was used for simultaneous visualization of versican and decorin. Sections were blocked with dual endogenous enzyme block that quench endogenous alkaline phosphatase and peroxidase. Versican were detected with an anti-versican rabbit polyclonal antibody (Atlas antibodies, Sweden, Stockholm), detected with a (HRP)-conjugated anti-rabbit secondary antibody and the non-permeable chromogen DAB. After a step with double staining blocking (making the first antibody inert to further staining by chemically destroying the antigenicity), decorin was visualized with an anti-decorin rabbit polyclonal antibody (Atlas antibodies), detected with an alkaline phosphatase (AP)-conjugated anti-rabbit secondary antibody and Permanent Red chromogen (Table 2). A single staining protocol (EnVision^TM^ Detection system, K5007, Dako) was used for visualization of biglycan, MMP-9, TIMP-3, fibronectin and EDA-fibronectin. Briefly, biglycan was detected by an anti-biglycan rabbit polyclonal antibody (Atlas antibodies, Stockholm, Sweden), anti MMP-9 by a rabbit polyclonal antibody (Dako, Glostrup, Denmark), anti TIMP-3 by a monoclonal mouse antibody (Millipore, Temecula, CA, USA), fibronectin by a monoclonal mouse antibody (Novocastra, Newcastle upon Thyne, UK), EDA-fibronectin by a polyclonal rabbit antibody (Abcam, Cambridge, UK) and secondary antibodies conjugated with peroxidase polymers. The immunohistochemistry protocols were performed using an automated immunohistochemistry robot (Autostainer, Dako). Sections were stained with Mayer’s haematoxylin for visualization of background tissue. Double stained slides were dried, immersed in xylene and mounted in Pertex (Histolab, Stockholm, Sweden) and single stained slides were dehydrated in alcohol and xylene and mounted in Pertex (Histolab).

*Immunohistochemical Staining of Fibroblasts*

After antigen retrieval and a blocking step (protein-blocking, X0909, Dako), sections were incubated overnight in 4°C with a rabbit anti-P4OH antibody (Atlas antibodies, Stockholm, Sweden) and immunoreactivity was visualised after 1 hour incubation in RT with a goat anti-rabbit Alexa-Flour 488-conjugated secondary antibody (1:200, Molecular Probes, Oregon, USA). Vimentin were detected after 1 hour incubation in RT with a mouse anti-vimentin (NovoCastra, Newcastle upon Tyne, UK) and visualized after incubation in RT for 1 hour with an Alexa-Flour 647-conjugated goat anti-mouse secondary antibody (1:200, Molecular Probes). α-SMA was detected with a mouse anti-αSMA antibody directly conjugated to Cy3 (Sigma-Aldrich, St. Louis, MO, US). Nuclei were detected by Hoechts 33342 and sections were mounted in TBS/glycerin and frozen until quantification.

*Immunohistochemical Staining of MMP-9 and Immune cells*

After antigen retrieval (high pH) and a blocking step (protein-blocking, X0909, Dako), sections were incubated with a rabbit anti-MMP9 antibody (Dako, Glostrup, Denmark) and immunoreactivity was visualised after 1 hour incubation in RT with a goat anti-rabbit Alexa-Flour 488-conjugated secondary antibody (1:200, Molecular Probes, Oregon, USA). Sections were then either incubated with anti-elastase (Dako, Glostrup, Denmark), or anti-CD68 (Dako, Glostrup, Denmark) and visualized after incubation in RT for 1 hour with an Alexa-Flour 647-conjugated goat anti-mouse secondary antibody (1:200, Molecular Probes). Nuclei were detected by Hoechts 33342 and sections were mounted in TBS/glycerin and frozen until quantification. See results in Figure S1 in the Online Supplement.

**Tissue Analysis**

***Quantification of Density of Versican, Decorin, Biglycan, MMP-9, TIMP-3.***

Stained slides were digitally scanned using ScanScope (Aperio, Vista, CA). All markers were quantified in blinded sections in central airways (bronchial biopsies) and alveolar parenchyma (transbronchial biopsies). The density (immunoreactivity per mm^2^ tissue area) of versican, decorin, biglycan, MMP9 and TIMP-1 and -3 as well as the tissue area in the walls of bronchi and in the alveolar septa was calculated using Visiomorph™ (Visiopharm, Hoersholm, Denmark). The image analysis program calculated the tissue area of the whole biopsy, excluding air spaces so that only tissue (i.e. airway epithelium, lamina propria and smooth muscle layer, or the alveolar septa) was measured. Glands were excluded from the analysis by manual detection.

***Quantification of Myofibroblasts***

The density of fibroblast was counted manually and calculated either as double positive for prolyl 4-hydroxylase (P4OH) (green: 488 nm) and vimentin (deep red: 647 nm) (fibroblasts) or triple positive for αSMA (red: 555 nm), P4OH (green: 488 nm) and vimentin (deep red: 647 nm) (myofibroblasts). The number of cells was related to total tissue area (cells/mm^2^) using Visiomorph™ (Visiopharm). Nuclei were detected by Hoechts 33342 that labels DNA (blue: 350 nm). All individual fibroblasts and myofibroblasts were analyzed in each bronchial and transbronchial biopsy. Sections were analyzed using NIS-elements AR 3.0 system (Nikon, Tokyo, Japan), a Nikon Eclipse 80i microscope, and a Nikon DS-Qi1Mc camera.

**Immunoassay for MMP-9 in BAL and Sputum**

Concentration of MMP-9 (active and pro), in bronchoalveolar lavage fluid (BAL) and sputum samples was measured by enzyme-linked immunosorbent assay (ELISA) in accordance to manufacturer’s instructions (MMP-9 ELISA DuoSet, R&D Systems, MN, USA). See results in Figure S2.

**RESULTS**

**Demographic and clinical characteristics**

In the group of patients with uncontrolled asthma, three patients had seasonal allergy and 13 patients had perennial allergy. Two patients were treated with leukotriene-receptor antagonist; three patients were treated with antihistamines and two with nasal steroids.


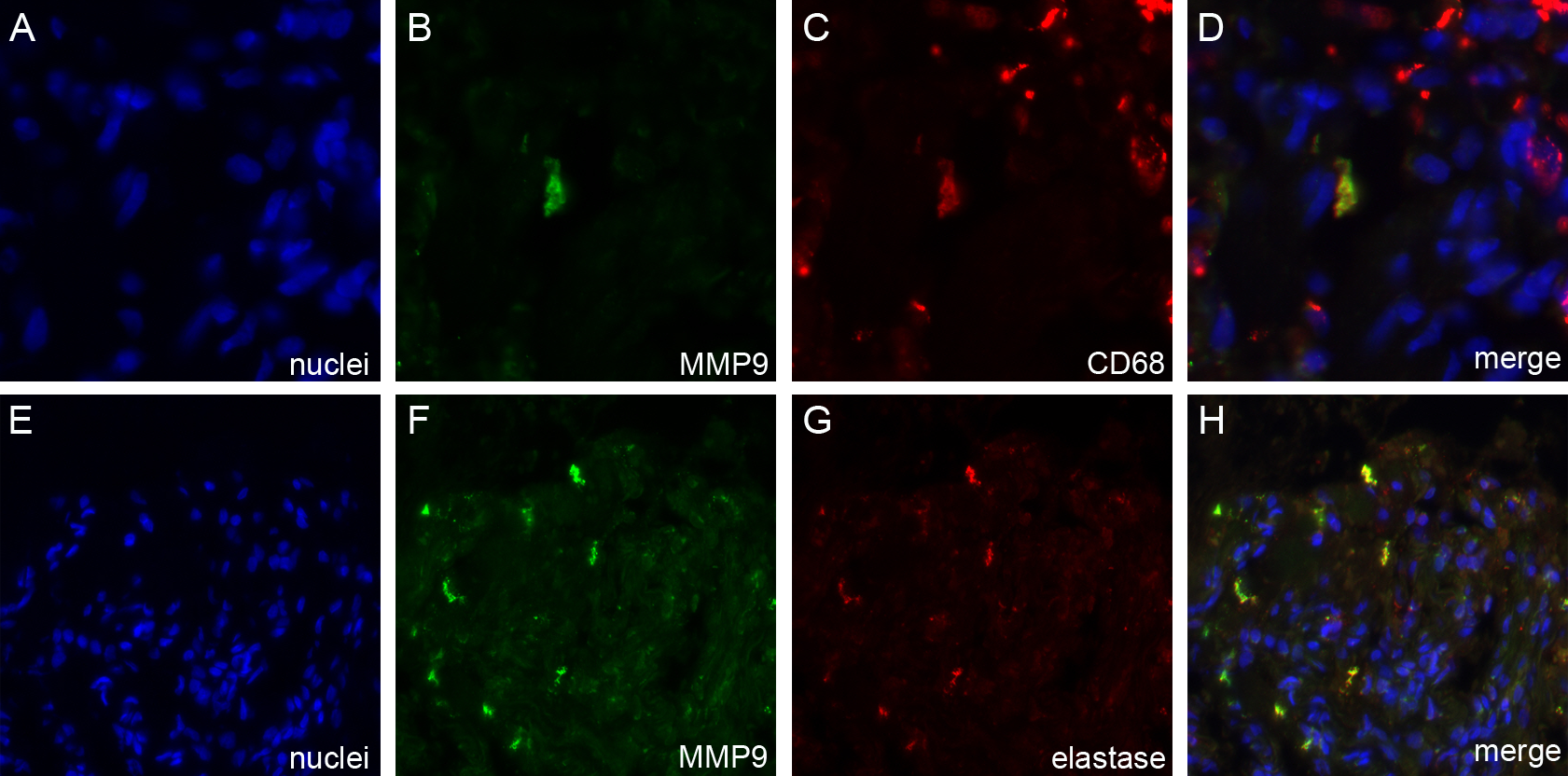


**Figure S1.** Double immunohistochemical staining of alveolar parenchyma of patients with uncontrolled asthma, showed that MMP-9 positive cells were mainly neutrophils (elastase positive) and macrophages (CD68 positive).

**Figure S2.** The concentrations of MMP-9 in BAL and sputum were measured in healthy controls and patients with controlled and uncontrolled asthma. No differences were found between controls or either group of asthmatics in BAL or sputum. The concentration of MMP-9 in sputum of patients with uncontrolled asthma correlated negatively with ACT-score (r_s_ = -0.58, p = 0.025), Table 2. The concentration of MMP-9 in BAL correlated positively with number of neutrophils in BAL from patients with uncontrolled asthma (r_s_ = 0.61, p = 0.017), Table 2.

**Figure S3.** Ratios of MMP-9/TIMP-3 in central airways and alveolar parenchyma of patients with controlled and uncontrolled asthma and healthy controls. Ratios were made by division of values of immunoreactivity per μm^2^ tissue.


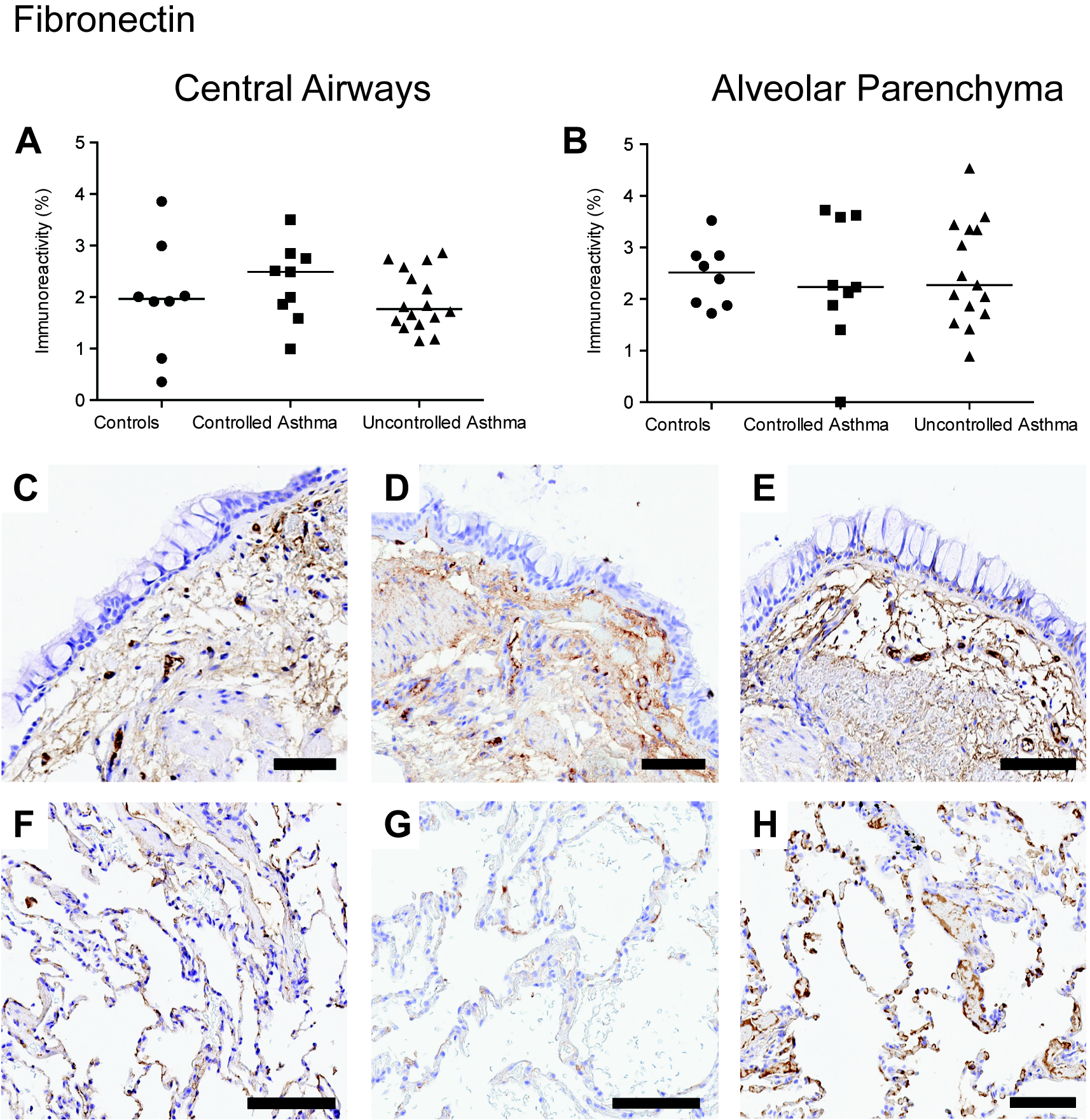


**Figure S4.** No difference in total fibronectin percentage area was found between healthy controls and asthmatics in either central airways or alveolar parenchyma, FigureS4 A-B. Representative micrographs of staining of fibronectin (brown) from controls (C, F) and patients with controlled asthma (D, G) and uncontrolled asthma (E, H) in bronchial (C-E) and transbronchial (F-H) biopsies. Scale bars: C and D = 50 μm, E-H = 100 μm.

**References**

1. Tufvesson E, Aronsson D, Bjermer L: **Cysteinyl-leukotriene levels in sputum differentiate asthma from rhinitis patients with or without bronchial hyperresponsiveness.** *Clin Exp Allergy* 2007, **37:**1067-1073.

2. Andersson CK, Bergqvist A, Mori M, Mauad T, Bjermer L, Erjefalt JS: **Mast cell-associated alveolar inflammation in patients with atopic uncontrolled asthma.** *J Allergy Clin Immunol* 2011, **127:**905-912 e901-907.

3. Tufvesson E, Aronsson D, Ankerst J, George SC, Bjermer L: **Peripheral nitric oxide is increased in rhinitic patients with asthma compared to bronchial hyperresponsiveness.** *Respir Med* 2007, **101:**2321-2326.

4. Tsoukias NM, George SC: **A two-compartment model of pulmonary nitric oxide exchange dynamics.** *J Appl Physiol* 1998, **85:**653-666.

5. Crapo RO, Morris AH, Gardner RM: **Reference spirometric values using techniques and equipment that meet ATS recommendations.** *Am Rev Respir Dis* 1981, **123:**659-664.

6. Aronsson D, Tufvesson E, Bjermer L: **Allergic rhinitis with or without concomitant asthma: difference in perception of dyspnoea and levels of fractional exhaled nitric oxide.** *Clin Exp Allergy* 2005, **35:**1457-1461.

| **Table S1. BAL cells** | | | |
| --- | --- | --- | --- |
| **Cell Type** | **Controls** | **Controlled Asthma** | **Uncontrolled Asthma** |
| Eosinophil | 1 (0.5-1) | 1 (1-2) | 0.5 (0-8) |
| Lymphocyte | 4 (2-14) | 4 (3-7) | 5 (2-15) |
| Macrophage | 82 (64-94) | 91 (89-94) | 90 (73-98) |
| Neutrophil | 3 (2-11) | 2 (2-5) | 1 (0.5-19) |
| Cells in the BAL from healthy controls (n=5), patients with controlled asthma (n=9) and uncontrolled asthma (n=16), were collected and stained with May-Grünwald and 6% Giemsa solution. Cellular profile was analyzed by counting the stained cells using a light microscope. | | | |

| **Table S2. Antibodies used for immunohistochemistry** | | | | |
| --- | --- | --- | --- | --- |
| **Primary antibody** | **Dilution** | **Antigen retrieval pretreatment** | **Clone and Manufacturer** | **Secondary antibody** |
| Polyclonal rabbit anti-Versican | 1:750 | High pH (Dako pretreatment) | Atlas Antibodies, Uppsala, Sweden | Envision, Dako K5361 |
| Polyclonal rabbit anti-Decorin | 1:1000 | High pH (Dako pretreatment) | Atlas Antibodies, Uppsala, Sweden | Envision, Dako K5361 |
| Polyclonal rabbit anti-Biglycan | 1:500 | High pH (Dako pretreatment) | Atlas Antibodies, Uppsala, Sweden | Envision, Dako K5007 |
| Polyclonal rabbit  anti MMP-9 | 1:100 | High pH (Dako pretreatment) | Dako, Glostrup, Denmark | Envision, Dako K5007 |
| Monoclonal mouse anti TIMP-3 | 1:1000 | High pH (Dako pretreatment) | Millipore, Temecula, CA, USA | Envision, Dako K5007 |
| Polyclonal rabbit anti-prolyl 4 OH | 1:4000 | Low pH (Dako pretreatment) | Atlas Antibodies, Uppsala, Sweden | Goat anti-rabbit AlexaF 488 |
| Monoclonal mouse anti-vimentin | 1:200 | Low pH (Dako pretreatment) | Novocastra, Newcastle upon Thyne, UK | Goat anti-mouse AlexaF 647 |
| Cy3 conjugated monoclonal mouse anti-α-SMA | 1:400 | Low pH (Dako pretreatment) | Sigma Aldrich Sweden, Stockholm, Sweden | Direct conjucated to Cy3 (AlexaF 555) |
| Monoclonal mouse anti-fibronectin | 1:4000 | High pH (Dako pretreatment) | Novocastra, Newcastle upon Thyne, UK | Envision, Dako K5007 |
| Polyclonal rabbit anti EDA-fibronectin | 1:1000 | High pH (Dako pretreatment) | Abcam, Cambridge, UK | Envision, Dako K5007 |
| Heat-induced antigen retrieval was performed in PT Link (Dako, Glostrup, Denmark). | | | | |
